# EPIMIC: A Simple Homemade Computer Program for Real-Time EPIdemiological Surveillance and Alert Based on MICrobiological Data

**DOI:** 10.1371/journal.pone.0144178

**Published:** 2015-12-14

**Authors:** Philippe Colson, Jean-Marc Rolain, Cédric Abat, Rémi Charrel, Pierre-Edouard Fournier, Didier Raoult

**Affiliations:** 1 Institut Hospitalo-Universitaire (IHU) Méditerranée Infection, Pôle des Maladies Infectieuses et Tropicales Clinique et Biologique, Fédération de Bactériologie-Hygiène-Virologie, Centre Hospitalo-Universitaire Timone, Assistance publique—hôpitaux de Marseille, 264 rue Saint-Pierre, 13385, Marseille, cedex 05, France; 2 Aix-Marseille Univ., Unité de Recherche sur les Maladies Infectieuses et Tropicales Emergentes (URMITE) UM 63 CNRS 7278 IRD 3R198 INSERM U1095, 27 boulevard Jean Moulin, 13385, Marseille, cedex 05, France; 3 Aix Marseille Université, IRD French Institute of Research for Development, EHESP French School of Public Health, EPV UMR D190 "Emergence des Pathologies Virales", Marseille, 13385, France; University Hospital San Giovanni Battista di Torino, ITALY

## Abstract

**Background and Aims:**

Infectious diseases (IDs) are major causes of morbidity and mortality and their surveillance is critical. In 2002, we implemented a simple and versatile homemade tool, named EPIMIC, for the real-time systematic automated surveillance of IDs at Marseille university hospitals, based on the data from our clinical microbiology laboratory, including clinical samples, tests and diagnoses.

**Methods:**

This tool was specifically designed to detect abnormal events as IDs are rarely predicted and modeled. EPIMIC operates using Microsoft Excel software and requires no particular computer skills or resources. An abnormal event corresponds to an increase above, or a decrease below threshold values calculated based on the mean of historical data plus or minus 2 standard deviations, respectively.

**Results:**

Between November 2002 and October 2013 (11 years), 293 items were surveyed weekly, including 38 clinical samples, 86 pathogens, 79 diagnosis tests, and 39 antibacterial resistance patterns. The mean duration of surveillance was 7.6 years (range, 1 month-10.9 years). A total of 108,427 Microsoft Excel file cells were filled with counts of clinical samples, and 110,017 cells were filled with counts of diagnoses. A total of 1,390,689 samples were analyzed. Among them, 172,180 were found to be positive for a pathogen. EPIMIC generated a mean number of 0.5 alert/week on abnormal events.

**Conclusions:**

EPIMIC proved to be efficient for real-time automated laboratory-based surveillance and alerting at our university hospital clinical microbiology laboratory-scale. It is freely downloadable from the following URL: http://www.mediterranee-infection.com/article.php?larub=157&titre=bulletin-epidemiologique (last accessed: 20/11/2015).

## Introduction

Infectious diseases (IDs) are major causes of morbidity and mortality worldwide [1–4]. Their surveillance is therefore critical to improve their diagnosis, prevention, clinical management and treatment [5–7]. Many surveillance systems target a limited number of IDs, and not throughout the whole year, but rather only for periods during which, classically, they are known to occur. These are important drawbacks that considerably limit the capability to detect “abnormal” events, including infections with unusual/unexpected features, and emerging/re-emerging diseases. Indeed, IDs are rarely predicted or modeled, as emphasized during recent epidemics [8–10]. In addition, the majority of ID surveillance tools do not lead to real-time detection and alert, preventing the rapid prioritization of public health threats and impairing the timely implementation of control strategies [7].

One of the surveillance approaches for IDs is syndromic surveillance that is based on non-specific markers available before confirmed diagnosis and that can be early and powerful surrogate indicators [11,12]. Several examples during past decades have highlighted that syndromic surveillance and warning systems could reveal major infections and outbreaks. These included the detection in 1976 of an unexplained mortality rise in Philadelphia, USA, which led to the discovery of *Legionella pneumophila* as a causative agent of pneumonia in humans [13]; or the warning concerning a few “abnormal” prescriptions of pentamidine in Los Angeles in 1981, which attracted attention on the first cases of acquired immunodeficiency syndromes [14].

Clinical microbiology laboratories represent a wealth of information, including data usable for syndromic surveillance consisting of numbers and types of clinical samples collected and of tests prescribed by clinicians, in addition to diagnoses [15,16]. In 2002, back from a stay in the USA for a mission on bioterrorism [17], one of the authors (DR) decided to implement a simple and versatile tool for the real-time systematic surveillance of IDs at Marseille university hospitals, based on data from our clinical microbiology laboratory. This homemade system surveys clinical samples, tests and diagnoses. We describe here its principle, properties and limits.

## Materials and Methods

### Laboratory setting

**Fig 1:**
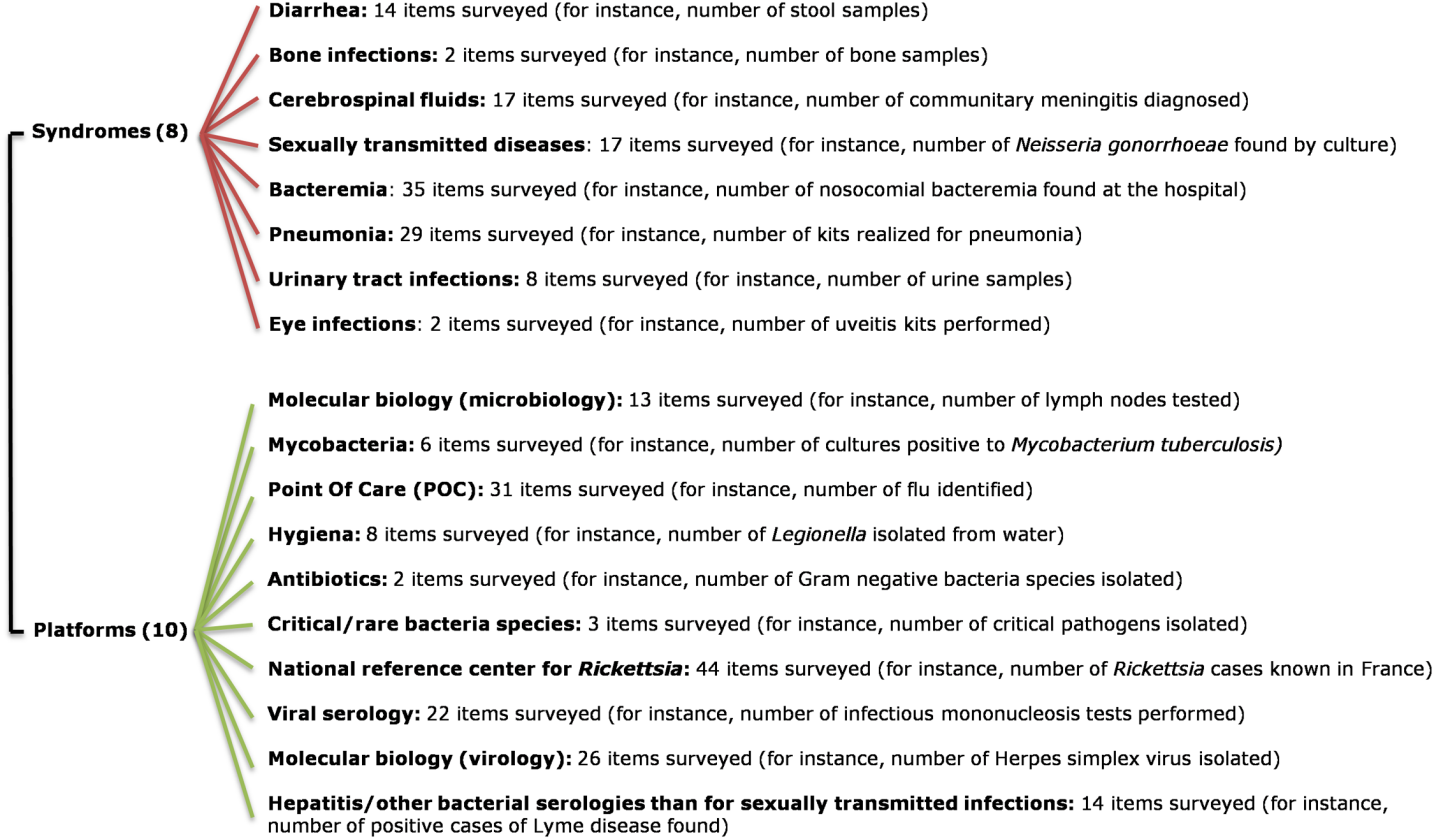
EPIMIC organization chart. Groups of items currently surveyed by EPIMIC, classified according to infectious syndromes or platforms based on specific technologies or dedicated to specific pathogens. See also S3 Table.

Between November 2002 and October 2013, we prospectively monitored the weekly numbers of clinical samples received, tests performed and positive and negative diagnoses obtained at the clinical microbiology laboratories of the four university hospitals of Marseille. Between 2002 and 2009, there were two core laboratories located in different hospitals. Since 2009, only one core laboratory remained that is located at Timone hospital, and two point of care laboratories were implemented in different hospitals. These three clinical microbiology laboratoires carry out all the microbiological analyses prescribed by clinicians in any of the four university hospitals (Fig 1). Marseille, the second largest French city, encompasses ≈850,000 inhabitants (http://www.insee.fr (last accessed: 20/11/2015); 2010). Its university hospitals comprise ≈4,000 beds and cumulate yearly ≈800,000 consultations and 790,000 days of hospitalisation [18]. Our clinical microbiology laboratory performs annually approximately 145,000 serological tests, 200,000 PCR and 220,000 cultures.

### Computer program operation

**Fig 2:**
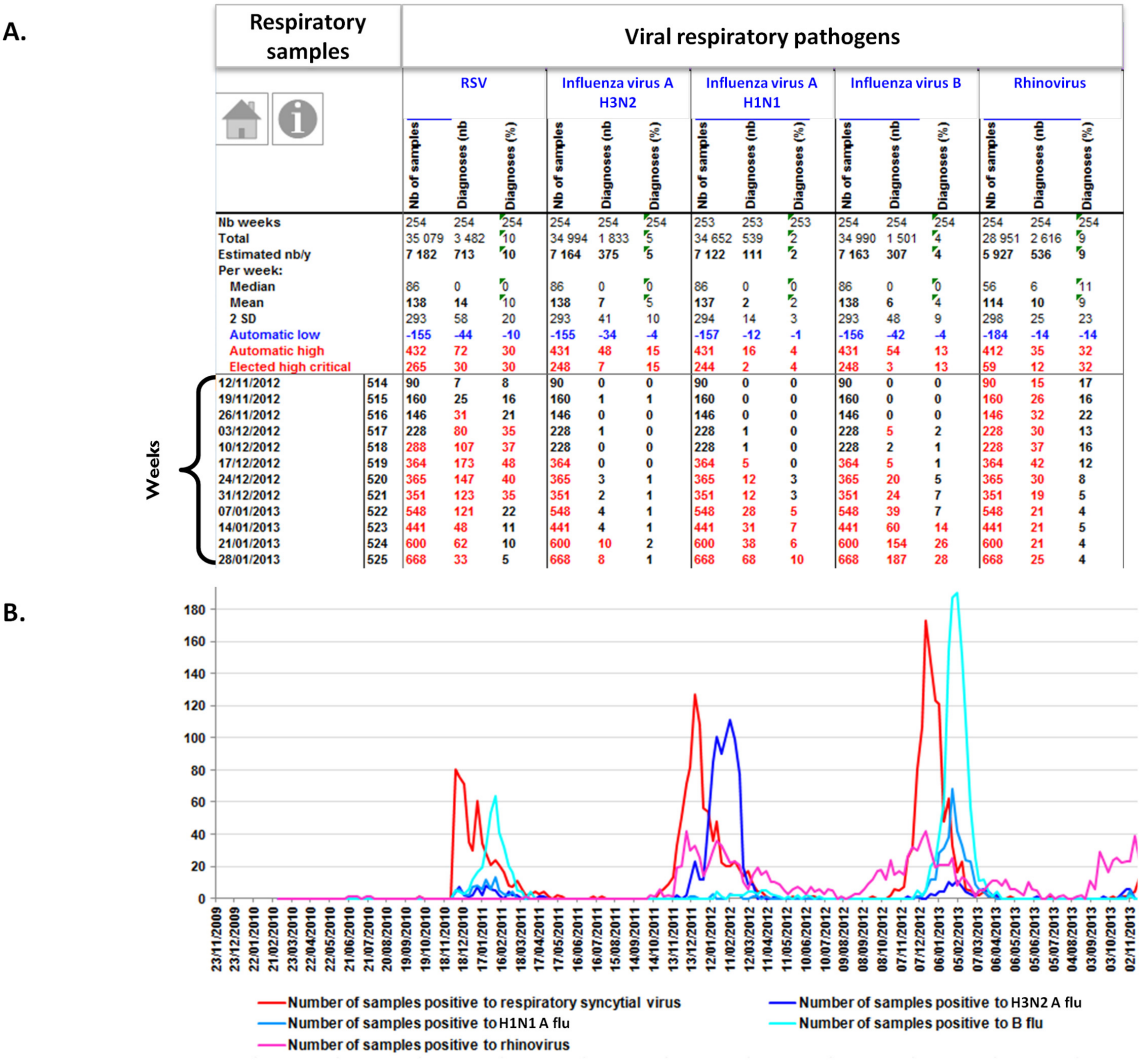
Examples of EPIMIC respiratory infection surveillance tables and plots. Table (top; A) shows counts of respiratory samples and viral diagnoses entered each week in an EPIMIC Microsoft Excel file; numbers in red font are those above the alert threshold corresponding to the mean plus 2 standard deviations calculated for historical data and shown in the top rows of the table (the critical threshold was adjusted here by discarding from historical data those corresponding to epidemic periods). Plot (bottom; B) shows trends of weekly numbers of samples positive for respiratory viruses. Nb, number; RSV, respiratory syncytial virus.

**Fig 3:**
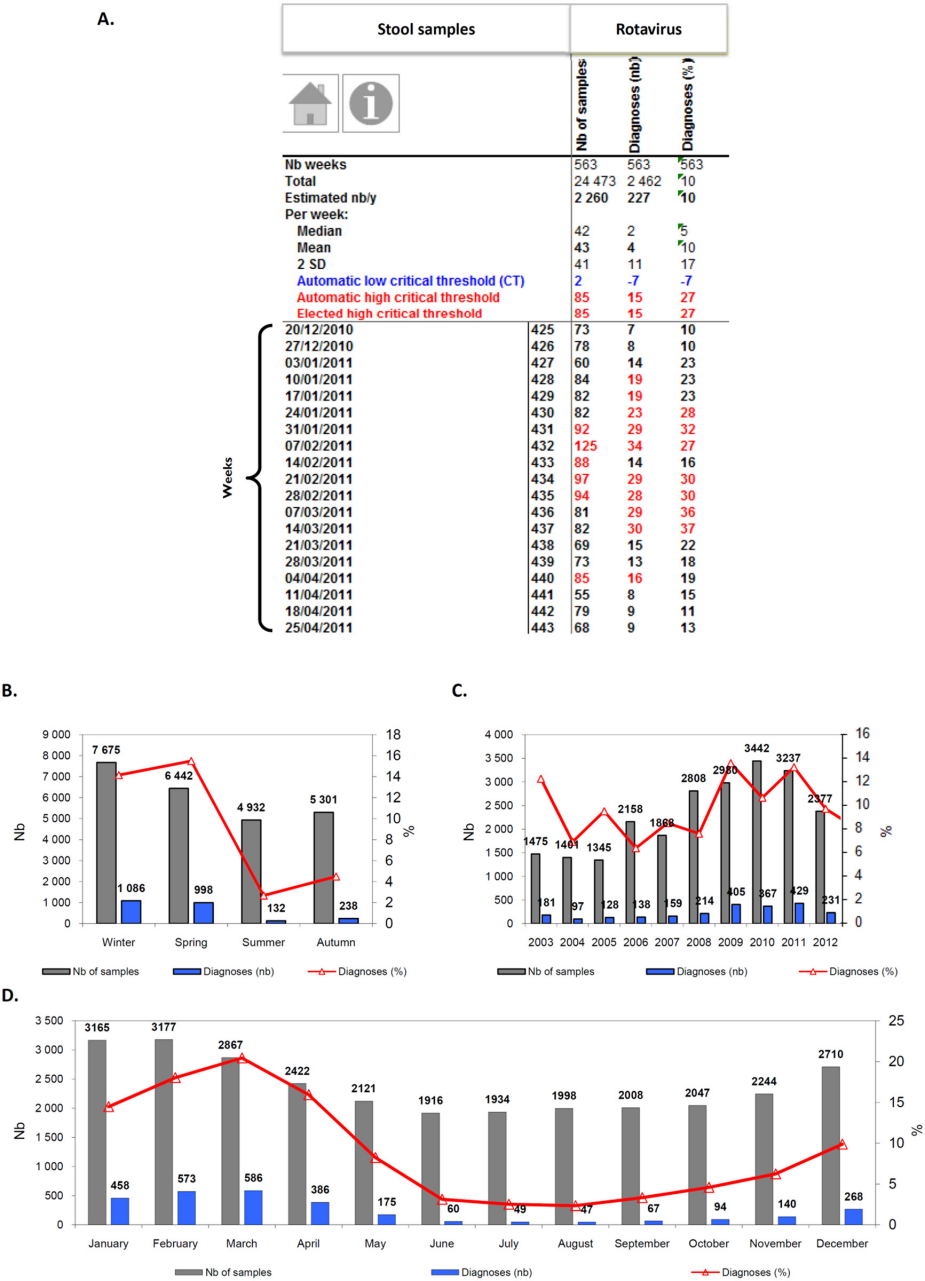
Examples of EPIMIC stool samples and rotavirus diagnoses surveillance tables and plots. Table (A) shows counts of stool samples and positive diagnoses of rotavirus entered each week into an EPIMIC Microsoft Excel file; numbers in red font are those above the alert threshold corresponding to the mean plus 2 standard deviations calculated for historical data and shown in the top rows of the table. Plots B, C and D show cumulated weekly numbers of stool samples received at our laboratory, of positive rotavirus diagnosis, along with the proportions of positive samples per season (B), year (C) and month (D). Nb, number.

Our homemade computer tool, named EPIMIC (for EPIdemiological surveillance and alert based on MICrobiological data) was implemented using the Microsoft Excel software. Data were split into several files accessible via a shared drive to any PC computer in the laboratory (S1 Table). Each of these files encompasses a dozen parameters, fitting the capability of our standard PC computers to open and run them; parameters from a given file are related to a given clinical syndrome or technological platform. These files can be accessed through hyperlinks from a Microsoft PowerPoint slide that presents our entire surveillance activity, which is split into various infectious syndromes or technological platforms (Fig 1). Laboratory data are collected weekly, either manually or automatically from our laboratory computer system, then entered manually into the different Microsoft excel files by a medical biology resident. Basically, triplets of numbers are entered, corresponding to weekly counts of clinical samples handled, tests performed and positive diagnoses, and proportions of positive diagnoses are automatically calculated; these are activity data and no patient record or information is entered. Each of the newly-entered weekly counts grows the set of historical data. Mean, standard deviation (SD) and mean±2 SD are automatically calculated for these historical data, and counts from the week are automatically compared to values corresponding to mean±2 SD. For instance, Fig 2A shows the numbers of respiratory samples tested and found positive for viral pathogens, and Fig 3A shows the numbers of stool samples tested and found positive for rotavirus. Finally, all counts are automatically plotted on graphs showing weekly, monthly, per season and yearly numbers of events as shown in Figs 2B and 3B–3D.

### Detection of abnormal events

An abnormal event corresponds to an increase above, or a decrease below threshold values calculated based on the mean of historical data plus or minus 2 SD, respectively. While entering weekly data, conditional formatting from the Excel software automatically changes the font to red if numbers are above the mean+2 SD and to blue if they are below the mean-2 SD. These automatically calculated thresholds can be replaced by others chosen by the user. Computed data are presented at least once a week during medical meetings, and interpreted by microbiologists. Confirmed alerts are reported to clinicians, and, depending on their nature, to a committee for the control of nosocomial infections, to the health regional agency, or to other French sanitary surveillance institutions.

### Statistical analysis of antibiotic-resistance surveillance data

Statistical analyses were performed for the surveillance of antibiotic-resistance patterns using linear models and the LOESS (locally weighted polynomial regression) regression curve to determine whether the proportion of isolated bacterial strains presenting a particular resistance profile monitored by EPIMIC significantly increased or decreased throughout the surveillance period. The tests were two-sided, p-values < 0.05 being considered as statistically significant, and were performed using the R program (Auckland, New-Zealand).

### Search for other laboratory-based surveillance systems for IDs

In order to compare EPIMIC to other laboratory-based surveillance systems, we identified other such systems through a PubMed search (URL: http://www.ncbi.nlm.nih.gov/pubmed) over the last 5 years using "laboratory-based surveillance" as keyword (S2 Table).

### Availability of the computer tool

A ready-to-use EPIMIC file can be freely downloaded from the University Hospital Institute (IHU) “Méditerranée Infection” foundation website (S1 Table; URL: http://www.mediterranee-infection.com/article.php?larub=157&titre=bulletin-epidemiologique (last accessed: 20/11/2015)).

## Results

### EPIMIC datasets

Between November 2002 and October 2013 (11 years), 293 items were surveyed weekly, including 38 clinical samples, 86 pathogens, 79 diagnosis tests, and 39 antibacterial resistance patterns (S3 Table). The mean duration of surveillance was 7.6 years (range, 1 month-10.9 years). A total of 108,427 Microsoft Excel file cells were filled with counts of clinical samples, and 110,017 cells were filled with counts of diagnoses. EPIMIC was used at our laboratory by 15 senior biologists and ≈30 residents in medical biology per year; the training period for each new person was approximately 10 min. In addition, as Microsoft Excel is a widely used and easy to operate software, EPIMIC could be created, run, maintained and repaired without the need of a resource person with high levels of computer skills.

**Fig 4:**
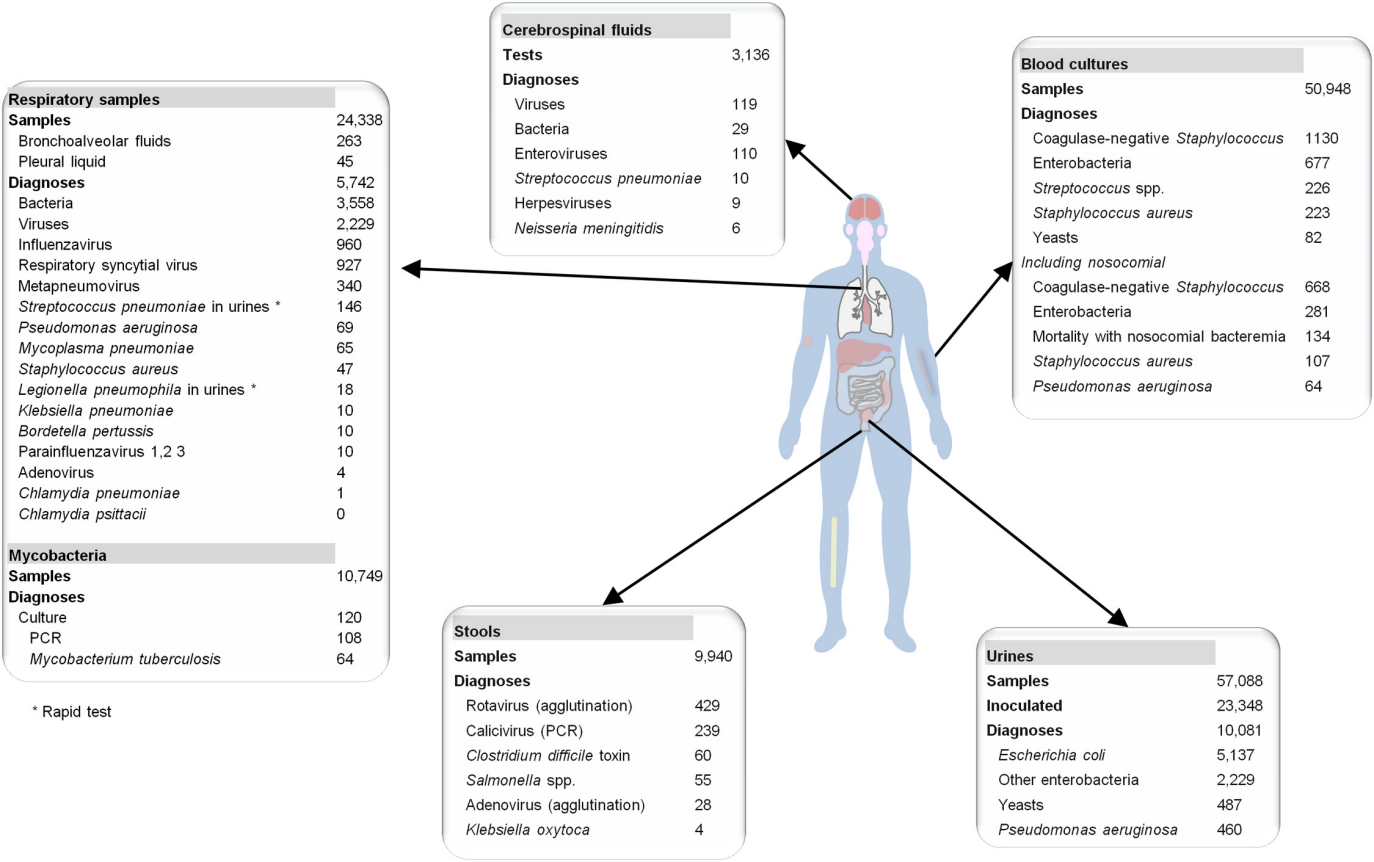
Examples of numbers of samples handled and positive diagnoses performed in 2011 at our laboratory.

Table 1 summarizes numbers of samples and diagnoses during the study period for the seven main types of samples surveyed by EPIMIC and the major pathogens diagnosed. A total of 1,390,689 samples were analyzed. Among them, 172,180 were found to be positive for a pathogen. Pathogens that were the most frequently diagnosed from respiratory samples, urine, stools, blood cultures and cerebrospinal fluids were respiratory syncytial virus (4,939 positive diagnoses), *E*. *coli* (42,874 strains), rotavirus (2,464 positive diagnoses), coagulase-negative *Staphylococcus* (7,006 strains) and enteroviruses (922 positive diagnoses), respectively. At a one-year scale, in 2011, the most numerous clinical samples received at our laboratory were urine samples (57,088), followed by blood cultures (50,948 samples) and respiratory samples (24,338) (Fig 4). *Escherichia coli* (5,137 strains) and coagulase-negative *Staphylococcus* (1,130 strains) were the bacterial species most frequently isolated from urine and blood, respectively. Regarding respiratory samples, influenza virus, respiratory syncytial virus and human metapneumovirus were the most frequently diagnosed viruses, representing 960, 927 and 340 cases, respectively, and *Pseudomonas aeruginosa* was the most frequently isolated bacterium, representing 69 cases. In addition, the pathogens by far the most frequently diagnosed from cerebrospinal fluids were enteroviruses (in 110 cases).

**Table 1:** Summary of the main types of clinical samples surveyed by EPIMIC and, by sample type, of main pathogens surveyed.

Sample type (surveillance period)	Total number of samples		Mean number of samples per week		Standard deviation of the number of samples per week		Main pathogens isolated from the samples	Total number of positive samples	Mean number of positive samples per week	Standard deviation of the weekly number of positive samples
	Tested	Positive	Tested	Positive	Tested	Positive				
Urine samples (from 04/11/2002 to 30/10/2013)	560 955	84 174	972	146	165	52	*Escherichia coli*	42,874	74	24
							*Pseudomonas aeruginosa*	4,007	7	3
Blood cultures (from 03/11/2003 to 30/10/2013)	496 891	33 619	937	63	158	26	Coagulase-negative *staphylococcus*	7,006	13	8
							*Staphylococcus aureus*	2,369	4	3
							*Streptococcus* sp.	2,175	4	2
Respiratory samples (from 11/11/2002 to 30/10/2013)	169 147	29 597	320	53	142	44	Respiratory syncytial virus	4,939	9	18
							Influenza virus	2,976	15	39
							*Pseudomonas aeruginosa*	584	1	1
							*Staphylococcus aureus*	531	1	1
Stool samples (from 04/11/2002 to 30/10/2013)	94 045	5 118	163	9	60	7	Rotavirus	2,464	4	6
							Calicivirus	661	3	4
							*Salmonella* sp.	384	1	1
							*Clostridium difficile*	633	2	3
Cerebrospinal fluid (from 04/11/2002 to 30/12/2013)	-	17 231	-	3	-	4	Enterovirus	922	2	3
							*Streptococcus pneumoniae*	78	<1	<1
							*Neisseria meningitidis*	48	<1	<1
Bone samples (from 04/11/2002 to 30/10/2013)	8 801	2 142	20	5	10	3	N.a.	N.a.	N.a.	N.a.
Ocular samples (from 04/11/2002 to 30/10/2013)	3 211	299	6	0,5	4	1	N.a.	N.a.	N.a.	N.a.

N.a., not available

### Examples of EPIMIC benefits and use

EPIMIC was efficient at detecting abnormal events for various IDs. The surveillance of clinical samples was found to be more precocious in some cases than that of diagnoses to detect a rise in some IDs, as the number of clinical samples exceeded the warning threshold before the number of diagnoses. This was the case for respiratory samples during fall 2009 and 2010, for cerebrospinal fluids during summer 2007, or for stool samples during fall 2007, summer 2011 and winter 2013 and 2014. EPIMIC also allowed known seasonalities to be visualized, for instance for influenza virus, respiratory syncytial virus or rotavirus infections (Figs 2 and 3). Nonetheless, the period and intensity of these infections were found to substantially vary according to the year, and unexpected features were observed, including a dramatically low incidence of influenza virus infections in 2010, following the 2009 H1N1 pandemic [8,9]. Moreover, EPIMIC revealed the seasonality of bloodstream infections caused by *Klebsiella pneumoniae* during the summer months, which was previously unknown [19].

Another example of abnormal event detected by EPIMIC was an increase in autochthonous hepatitis E diagnosed during early 2011 [20]. This rise was associated with consumption of raw pig liver sausage (more traditionally eaten around Christmas and New Year eve) in 55% of cases, and the emergence in our geographical area of genotype 4 HEV infections, formerly found mainly in China, not in Europe. EPIMIC also revealed during early 2011 an abnormal increase in Group A *Streptococcus* (GAS) infections [21]. The ensuing investigations revealed that these infections mostly affected children, and as a study in UK concurrently described cases of infections with influenza B and invasive GAS [22], we further noted that 23 of 74 samples (31%) testing positive for GAS infection also tested positive for influenza virus. In addition, EPIMIC allowed the first report of a rise in 2012 of sexually transmitted diseases, including gonorrhea, syphilis and primary HIV infection [23], and the same year, a 71% increase of the incidence of parvovirus B19 infections was observed compared to the average yearly incidence reported during the ten previous years (2002–2011) [24]. Moreover, EPIMIC allowed the rapid detection of hypervirulent and highly transmissible *Clostridium difficile* clone 027 in our geographical area in 2013 [25].

**Fig 5:**
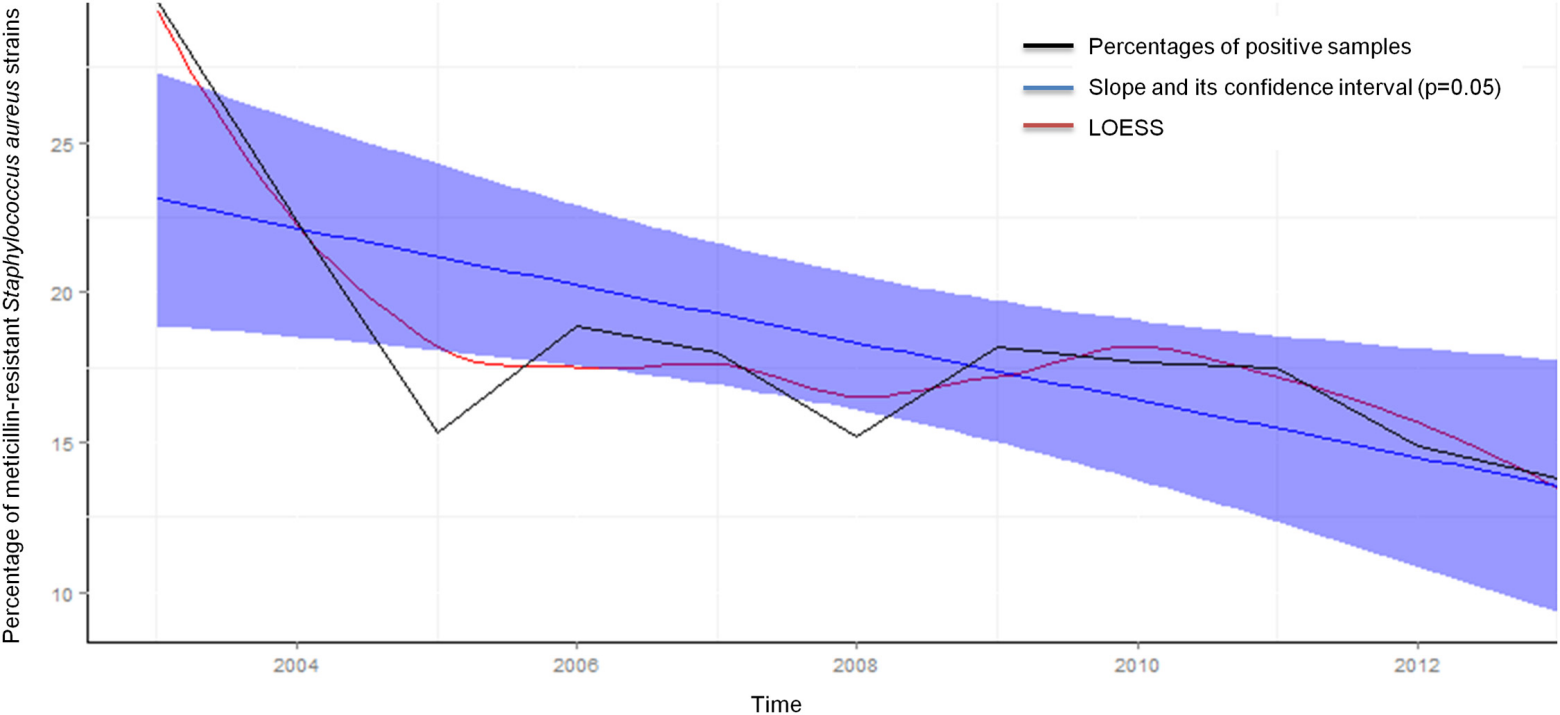
EPIMIC methicillin-resistant *Staphylococcus aureus* surveillance plot. The purple envelope represents the 95% confidence interval of the blue slope.

Regarding antibiotic-resistance, EPIMIC also identified an abnormal increase between December 2010 and April 2011 in the number of *Acinetobacter baumannii* strains exhibiting a carbapenem-resistant profile at Marseille university hospitals [26]. Moreover, EPIMIC allowed us to survey specific antibiotic-resistance profiles for various bacterial species defined as critical pathogens. This allowed, for instance, to observe that the weekly percentage of samples positive for *S*. *aureus* strains resistant to methicillin decreased significantly by 0.0099% on average throughout the study period (from 33.4% for the first week of December 2003 to 13.5% for the last week of December 2013, p < 10^−5^) (Fig 5). This finding is consistent with those recently reported in France and worldwide, and described in our institution for invasive methicillin-resistant *S*. *aureus* infections [27]. EPIMIC was also contributive in the retrospective analysis of intrinsic colistin-resistant bacteria in Marseille university hospitals in the context of an increasing burden of urinary tract infections [28]. Overall, between June 2013 and October 2014 (17 months), 0.46 non-recurrent alert 23 on abnormal events were detected per week.

Finally, EPIMIC was an educational tool as it showed the infectious syndromes and pathogens most frequently encountered at university hospitals of Marseille and in our geographical area to ≈200 students who stayed each year in our clinical microbiology laboratory for periods ranging from several days to several semesters.

### Comparison with other laboratory-based surveillance tools

A total of 76 other laboratory-based surveillance systems were identified through a PubMed search over the last 5 years (S2 Table), in Europe (n = 31; 41%) America (19), Asia (11), Africa (7), the Middle East (2) and the Pacific region (1); 5 systems (7%) were implemented for the purpose of global surveillance. Among these systems is the one implemented at the country-scale by the Health Protection Agency in England and Wales since the early 1990s, which counts infectious pathogens detected by hospital and specialist laboratories, and allowed trends for various pathogens to be described over long periods in England and Wales [16,29,30]. Amongst the 76 systems, 34 (45%) surveyed bacteria, 14 surveyed viruses, 9 surveyed yeasts and 2 surveyed parasites; for 17 (22%), targeted pathogens were not identified. Almost half (n = 36) of these 76 surveillance systems only surveyed one pathogen or topic (e.g., nosocomial infection, antimicrobial resistance, or invasive diseases). Nine systems (12%) surveyed between 2 and 13 pathogens or topics. Finally, 31 systems (41%) surveyed an undefined number of pathogens or topics. In contrast, during the study period EPIMIC surveyed 293 pathogens or topics. The mean (±standard deviation) duration of surveillance of the 76 surveillance systems was 10±10 years (range, 1–60 years), whereas mean duration of surveillance with EPIMIC was 11 years. Finally, only one third (25) of the 76 laboratory-based surveillance systems surveyed pathogens in real-time, and in a large majority of cases they focused on a single pathogen. By contrast, EPIMIC allowed the real-time surveillance of our entire clinical microbiology laboratory dataset.

## Discussion

EPIMIC was implemented in our clinical microbiology laboratory to allow the automatic and in real-time detection of any abnormal events related to IDs, assuming that they are rarely predictable and modeled [8]. Over an 11-year period, EPIMIC appeared as a simple, versatile and scalable tool that could be applied to any infectious syndrome and pathogen, and that was capable of managing a considerable amount of data at our clinical microbiology laboratory-scale. Moreover, our tool was efficient for automated real-time monitoring of IDs through both syndromic and traditional surveillance [6,7]. Thus, EPIMIC, in addition to detecting known seasonalities or expected events related to IDs, also identified abnormal events, including unexpected outbreaks and unknown seasonal phenomena [19,21,25]. These findings allowed us to report not only to clinicians from our institution, but also to regional and national institutions, and several of these findings were worthy of publication. EPIMIC was also an interesting educational tool for students, through objective assessment of the actual incidence and prevalence of IDs and pathogens.

Other automated laboratory-based surveillance systems were described as efficient to identify rises in IDs. Nevertheless, compared to these systems, EPIMIC continuously surveys and alerts on a more comprehensive dataset including clinical samples and tests, and not only pathogens. Moreover, EPIMIC does not focus on specific infectious threats during specific periods but performs surveillance without *a priori*, which is a prerequisite to detect unexpected events. In addition, historical data in EPIMIC are available over more than a decade, which is a longer duration than for most of the other systems. Importantly, EPIMIC automatically generates weekly alerts that are managed in real-time. Finally, our surveillance tool is user-friendly and can be used by any microbiologist as it operates using Microsoft Excel and requires no specific computer skills. Over the study period, EPIMIC was used by ≈300 residents and biologists trained within minutes. Furthermore, it can be implemented in any setting including unsophisticated ones because it can operate using basic PC computers with no specific cost. Thus, EPIMIC can be shared easily; a ready-to-use EPIMIC file is freely-available from our institution website.

Some limits of our surveillance computer tool are, notwithstanding, related to its absence of sophistication. Thus, some data are collected manually and all data are entered manually. Such human interventions can generate errors and false alerts, lowering the specificity of the surveillance system. Also, the statistical method used to set alert thresholds (based on the mean ±SD) is the same for all surveyed data, regardless of their amounts and variations during the year, and we are aware that such a global approach may not be the most appropriate in all cases [16,31,32]. Finally, the capabilities of EPIMIC in terms of performance and scalability are now limited in view of growing data and needs in our laboratory. As the development of epidemiological surveillance of IDs is one of the objectives of IHU Méditerranée Infection foundation, the introduction of new tools is on-going in collaboration with epidemiologists and computer scientists. Computer resources are expanding considerably; detection methods and alert thresholds will be optimized and adapted according to the data, and alert statements will be displayed continuously, available remotely, and transferred automatically to referents. However, EPIMIC might be useful for other laboratories in various settings, including in cases of limited computer resources.

In conclusion, we implemented an automated surveillance system that showed effective for 11 years to detect in real-time true abnormal events linked to infections and trigger alerts. The use of such epidemiological surveillance tools should be extended to gain a better knowledge of infectious diseases and improve their diagnosis, prevention, control and treatment.

## Supporting Information

S1 Table A ready-to-use EPIMIC file as freely-available from our institution website (http://www.mediterranee-infection.com/article.php?larub=157&titre=bulletin-epidemiologique (last accessed: 20/11/2015)). (XLS)

S2 Table Main features of laboratory-based surveillance systems identified through a PubMed search over the last 5 years. (XLSX)

S3 Table Items surveyed by EPIMIC. Items were classified according to infectious syndromes or platforms based on specific technologies or dedicated to specific pathogens.
(XLSX)
